# Comparison of Different Reverse Transcriptase–Polymerase Chain Reaction–Based Methods for Wastewater Surveillance of SARS-CoV-2: Exploratory Study

**DOI:** 10.2196/53175

**Published:** 2024-08-19

**Authors:** Annika Länsivaara, Kirsi-Maarit Lehto, Rafiqul Hyder, Erja Sinikka Janhonen, Anssi Lipponen, Annamari Heikinheimo, Tarja Pitkänen, Sami Oikarinen, WastPan Study Group

**Affiliations:** 1 Faculty of Medicine and Health Technology Tampere University Tampere Finland; 2 Expert Microbiology Unit Finnish Institute for Health and Welfare Kuopio Finland; 3 Department of Food Hygiene and Environmental Health Faculty of Veterinary Medicine University of Helsinki Helsinki Finland; 4 Finnish Food Authority - Ruokavirasto Seinäjoki Finland

**Keywords:** wastewater surveillance, surveillance systems, SARS-CoV-2, COVID-19, wastewater, surveillance, Finland, monitoring, detection, low-resource settings, RNA, spatial, temporal changes, reverse transcription droplet digital polymerase chain reaction, quantitative reverse transcription polymerase chain reaction, reverse transcription strand invasion based amplification

## Abstract

**Background:**

Many countries have applied the wastewater surveillance of the COVID-19 pandemic to their national public health monitoring measures. The most used methods for detecting SARS-CoV-2 in wastewater are quantitative reverse transcriptase–polymerase chain reaction (RT-qPCR) and reverse transcriptase–droplet digital polymerase chain reaction (RT-ddPCR). Previous comparison studies have produced conflicting results, thus more research on the subject is required.

**Objective:**

This study aims to compare RT-qPCR and RT-ddPCR for detecting SARS-CoV-2 in wastewater. It also aimed to investigate the effect of changes in the analytical pipeline, including the RNA extraction kit, RT-PCR kit, and target gene assay, on the results. Another aim was to find a detection method for low-resource settings.

**Methods:**

We compared 2 RT-qPCR kits, TaqMan RT-qPCR and QuantiTect RT-qPCR, and RT-ddPCR based on sensitivity, positivity rates, variability, and correlation of SARS-CoV-2 gene copy numbers in wastewater to the incidence of COVID-19. Furthermore, we compared 2 RNA extraction methods, column- and magnetic-bead–based. In addition, we assessed 2 target gene assays for RT-qPCR, N1 and N2, and 2 target gene assays for ddPCR N1 and E. Reverse transcription strand invasion-based amplification (RT-SIBA) was used to detect SARS-CoV-2 from wastewater qualitatively.

**Results:**

Our results indicated that the most sensitive method to detect SARS-CoV-2 in wastewater was RT-ddPCR. It had the highest positivity rate (26/30), and its limit of detection was the lowest (0.06 gene copies/µL). However, we obtained the best correlation between COVID-19 incidence and SARS-CoV-2 gene copy number in wastewater using TaqMan RT-qPCR (correlation coefficient [CC]=0.697, *P*<.001). We found a significant difference in sensitivity between the TaqMan RT-qPCR kit and the QuantiTect RT-qPCR kit, the first having a significantly lower limit of detection and a higher positivity rate than the latter. Furthermore, the N1 target gene assay was the most sensitive for both RT-qPCR kits, while no significant difference was found between the gene targets using RT-ddPCR. In addition, the use of different RNA extraction kits affected the result when the TaqMan RT-qPCR kit was used. RT-SIBA was able to detect SARS-CoV-2 RNA in wastewater.

**Conclusions:**

As our study, as well as most of the previous studies, has shown RT-ddPCR to be more sensitive than RT-qPCR, its use in the wastewater surveillance of SARS-CoV-2 should be considered, especially if the amount of SARS-CoV-2 circulating in the population was low. All the analysis steps must be optimized for wastewater surveillance as our study showed that all the analysis steps including the compatibility of the RNA extraction, the RT-PCR kit, and the target gene assay influence the results. In addition, our study showed that RT-SIBA could be used to detect SARS-CoV-2 in wastewater if a qualitative result is sufficient.

## Introduction

Wastewater reflects the circulation of microbes in the population in certain sewerage network areas and can be used to evaluate the circulation of various pathogens [1]. SARS-CoV-2 RNA is excreted in the feces of infected individuals [2], and the spatial and temporal changes of the COVID-19 pandemic can therefore be studied from wastewater [3-6]. The need for surveillance of SARS-CoV-2 in wastewater has been demonstrated by the fact that the estimated spread of COVID-19 based on wastewater surveillance has been much higher than would be expected based on clinical cases showing the actual spread of the virus [7]. In addition, it has been shown that wastewater monitoring of SARS-CoV-2 can be used to estimate new hospital and intensive care unit admissions 2-8 days ahead of time [8]. Furthermore, wastewater provides an easy and cost-efficient pooled sample matrix. A national wastewater surveillance system for SARS-CoV-2 has been implemented in several countries, the information from which is used in public health decision-making. The Centers for Disease Control and Prevention has started national wastewater surveillance in the United States [9], and the European Commission has called for the systematic surveillance of SARS-CoV-2 in wastewater in the European Union [10]. Furthermore, the European Commission has suggested that member states start surveilling influenza A and poliovirus from wastewater [11]. For the surveillance to be reliable, the use of a suitable method and careful optimization and validation of the method is necessary.

For the wastewater surveillance of SARS-CoV-2, both quantitative reverse transcriptase–polymerase chain reaction (RT-qPCR) and reverse transcriptase–droplet digital polymerase chain reaction (RT-ddPCR) are used. To date, comparisons between the 2 methods have been made regarding SARS-CoV-2 wastewater surveillance, although they have produced conflicting results. A previous study conducted during low COVID-19 incidence showed ddPCR to be more prone to inhibitors than RT-qPCR, to have a higher limit of detection (LOD), and to estimate lower gene copy (GC) numbers of the virus [12]. In contrast, Ahmed et al [13] discovered ddPCR to have a higher positivity rate and lower LOD than qPCR. Similarly, Ciesielski et al [14] noted RT-ddPCR to be more sensitive and to have a lower LOD than qPCR. The higher detection rate, sensitivity, and precision of ddPCR were also noted by Flood et al [15]. Lucansky et al [16] discovered ddPCR to be more specific and sensitive than qPCR. On the other hand, Boogaerts et al [17] found ddPCR and qPCR to be comparable in sensitivity. Previous studies have also noted differences in results when using different RNA extraction methods [18,19] and target gene assays [13,15,20]. In 2023, the incidence of COVID-19 has been on a downward trend [21]. As the amount of SARS-CoV-2 in wastewater declines, the need for a sensitive detection method increases. As previous results on the performance of polymerase chain reaction (PCR)–based detection methods have been conflicting, further research is needed.

RT-qPCR and RT-ddPCR each require extensive resources and expertise. In addition to identifying the most sensitive method to detect SARS-CoV-2 in wastewater, there is also a need for methods that require fewer resources and are easier to use. For fast and resource-saving analysis of SARS-CoV-2 in wastewater, 1 option is reverse transcription strand invasion-based amplification (RT-SIBA), a qualitative method based on the isothermal amplification of nucleic acids. Compared to PCR, RT-SIBA is faster, and the instruments required are less complex than they are for methods that require thermal cycling. It has previously been used to detect viruses, such as respiratory syncytial virus, influenza type A and B viruses, and rhinovirus, in clinical specimens [22-24]. According to our knowledge, there have been no studies of RT-SIBA to detect SARS-CoV-2 RNA in wastewater, and its use in the detection of SARS-CoV-2 for diagnostic use has been reported once [25]. Previously, 1 isothermal amplification method, loop-mediated isothermal amplification (RT-LAMP), was used to detect SARS-CoV-2 in wastewater, but the method was shown to be 20 times less sensitive than RT-ddPCR [26].

The aim of this study was to assess the performance of RT-qPCR and RT-ddPCR approaches for the wastewater-based surveillance of SARS-CoV-2. Furthermore, we compared 2 RNA extraction methods and different target genes to investigate the impact of alterations in the analysis pipeline on the detection of SARS-CoV-2 in wastewater-based surveillance. We assessed the sensitivity of the methods through their limit of detection and positivity rate and the repeatability of the methods through their intra- and interassay variability. We also explored the correlation between the amount of SARS-CoV-2 in wastewater, as determined by different methods, and the incidence of COVID-19. The samples were collected during low incidence of COVID-19 to test the methods’ sensitivity and performance in detecting a low amount of SARS-CoV-2. Furthermore, we tested an RT-SIBA assay for the qualitative detection of SARS-CoV-2 for wastewater surveillance in low-resource settings.

## Methods

### Wastewater Sampling

The samples used in this study are a part of the WastPan project conducted in collaboration with Tampere University, the Finnish Institute for Health and Welfare, and the University of Helsinki between 2020 and 2023. The project aims to develop tools for the wastewater-based surveillance of pathogens and antimicrobial resistance genes. This study includes 10 wastewater treatment plants (WWTPs) located in Espoo (Suomenoja WWTP, 390,000 inhabitants), Helsinki (Viikinmäki WWTP, 860,000 inhabitants), Kuopio (Lehtoniemi WWTP, 91,000 inhabitants), Lappeenranta (Toikansuo WWTP, 63,000 inhabitants), Oulu (Taskila WWTP, 200,000 inhabitants), Pietarsaari (Alheda WWTP, 31,000 inhabitants), Rovaniemi (Alakorkalo WWTP, 55,000 inhabitants), Seinäjoki (Seinäjoen keskuspuhdistamo WWTP, 55,000 inhabitants), Tampere (Viinikanlahti WWTP, 200,000 inhabitants), and Turku (Kakolanmäki WWTP, 300,000 inhabitants). The samples used in this paper were collected on February 22, 2021; March 21, 2021; and December 12, 2021. In addition, samples were collected from Espoo and Helsinki on May 17, 2021. From 24-hour composite samples of influent untreated wastewater, a fraction of 1 L was shipped in cool boxes within 24-48 hours of sampling. After arrival, the samples were frozen and kept in a –80 °C freezer before analysis [27].

The performance of the methods was tested using samples collected at various COVID-19 incidences in the WWTP areas. The wastewater samples were classified into 3 groups according to the incidence of COVID-19, based on the reported cases in the Finnish National Infectious Disease Register by the Finnish National Institute for Health and Welfare. Incidence was measured in terms of COVID-19 cases per 100,000 inhabitants over a 7-day period of the sampling week in the city in which the WWTP was located (incidence=cases/population of the WWTP city × 100,000). The low-incidence group (n=12) included samples collected at a time with less than 50 cases, the medium group 50-200 cases (n=9), and the high group over 200 cases (n=9) of COVID-19 per 100,000 inhabitants per week. Out of the 12 low-incidence group samples, 3 were collected when the incidence of COVID-19 was 0.

### Concentration of Wastewater Samples

Frozen samples were thawed in a refrigerator and then concentrated immediately, according to the method described by Hokajärvi et al [28]. First, a 100-mL aliquot of the 1 L original sample was melted in the refrigerator for the analysis. Immediately after the sample had melted, interfering particles, such as debris, were removed with centrifugation at 4654 ×g for 30 minutes without a brake. The supernatant was then concentrated using a Centricon Plus-70 centrifugal ultrafilter with a cutoff of 100 kDa (Millipore) and centrifuged at 3500 ×g for 15 minutes. The concentrate was collected by centrifugation at 1000 ×g for 2 minutes.

### RNA Extraction

RNA extraction was performed immediately after concentration. All samples were extracted with a PerkinElmer chemagic Viral DNA/RNA 300 (Wallac Oy), and the samples collected in February 2021, March 2021, and May 2021 were also extracted using a Qiagen QIAamp Viral RNA mini kit (Qiagen). Both extractions were carried out according to the manufacturer’s protocols. The sample volume was 300 µL for the chemagic RNA extraction kit and 140 µL for the QIAamp RNA extraction kit. The elution volume was 100 µL for the chemagic extraction and 60 µL for the QIAamp extraction. The samples were each extracted twice in the same extraction run to produce enough eluate for the method comparisons. The 2 eluates of the same sample were then mixed into 1 sample, which was used for all the detection methods. All the detection methods were performed from the same sample at the same time to enable equal comparison.

### SARS-CoV-2 Synthetic RNA Control

A 10-fold standard dilution series (1-10,000 copies/µL) of SARS-CoV-2 synthetic RNA control (Codex DNA) was run on each qPCR plate as a positive control to quantify the SARS-CoV-2 GCs. An RNA control was also used to calculate the LOD for each method.

### RT-qPCR

RT-qPCR was performed using 2 different RT-qPCR kits, TaqMan Fast Virus 1-Step Mastermix (TaqMan RT-qPCR; Applied Biosystems by Thermo Fisher Scientific) and Qiagen QuantiTect Probe RT-PCR (QuantiTect RT-qPCR; Qiagen), and the CDC N1 and N2 primer-probe sets. The Sarbeco E-gene primer-probe set [29] was also tested during the optimization of the RT-qPCR methods; we noted that most samples were nondetects, and thus, the E target gene assay was rejected from the study. The sequences of the primers and probes as well as the cycling conditions for all the RT-qPCR reactions are shown in the supplementary material (Tables S1-S3 in Multimedia Appendix 1). The specificity and sensitivity of the primers and probes were validated with the Quality Control for Molecular Diagnostics panel. Cross-reactivity to endemic coronaviruses 229E and OC43 was also tested using the panel, and no cross-reactivity was found. The TaqMan RT-qPCR was performed according to the manufacturer’s protocol using a total volume of 25 µL. The reaction mixture for the TaqMan N1 and N2 assays included 6.25 µL of the TaqMan Fast Virus 1-step mastermix, 200 nM forward primer, 200 nM reverse primer, 200 nM probe, and 5 µL template. The QuantiTect RT-qPCR had been previously optimized for clinical samples using a total volume of 10 µL and a template volume of 2 µL; initially, these volumes were used. The reaction mixture for the QuantiTect N1 gene assay included 5 µL of the QuantiTect RT mastermix, 900 nM forward primer, 900 nM reverse primer, 200 nM probe, 1 µL QuantiTect RT mix, and 2 µL template. The reaction mixture for the QuantiTect N2 gene assay included 5 µL of the QuantiTect RT mastermix, 300 nM forward primer, 900 nM reverse primer, 200 nM probe, 1 µL QuantiTect RT mix, and 2 µL template. In addition, the December 2021 samples were analyzed with the QuantiTect RT-qPCR assay using the manufacturer-recommended total volume of 25 µL and template volume of 5 µL while using the same primer and probe concentrations to evaluate the effect of the template volume. Negative control for RNA extraction and qPCR negative and positive controls were run on each plate. The samples were run in triplicate. Repeatability (intra-assay variation) was analyzed among the triplicates, and reproducibility (interassay variation) was determined using 3 different mastermix reactions, for a total of 9 repetitions. The runs were performed on the Applied Biosystems QuantStudio 5 Real-Time PCR System.

A sample was considered successfully amplified and positive if its Ct-value was below 40. In addition, for the quantification of the GCs, the 95% LOD was calculated using regression probit analysis [30]. The first 4 different concentrations of the SARS-CoV-2 synthetic RNA control (Codex DNA) were replicated 12 times, after which the proportion of positive reactions (probability of detection) was calculated. The probability of detection was transformed into probability units by the inverse of the normal cumulative distribution, and the concentrations were then transformed into base 10 logarithms. The probability units were plotted against the base 10 logarithms of the concentrations. Finally, the 95% LOD was calculated by solving the regression equation, where y=probability unit=1.64 (probability unit 1.64 equates to 95% probability). The efficiency, standard curve slope, standard curve intercept, and *R*² of each RT-qPCR assay are reported in Table S4 in Multimedia Appendix 1.

### RT-ddPCR

RT-ddPCR was performed using the Bio Rad One-Step RT-ddPCR Advanced Kit for Probes (RT-ddPCR; Bio Rad). Preliminary testing of the CDC N1, N2, and N3 primer-probe sets [31] and the Sarbeco E-gene primer-probe set [29] showed that the CDC N1 and the Sarbeco E-gene primer-probe sets were the most sensitive (data not shown). The sequences of the primers and probes as well as the cycling conditions for all the RT-ddPCR reactions are shown in the supplementary material (Tables S1 and S5 in Multimedia Appendix 1). RT-ddPCR was performed according to the manufacturer’s protocol using a template volume of 4 µL and a total volume of 20 µL. The reaction mixture for the N1 and E assays included 5.5 µL of supermix, 100 nM forward primer, 100 nM reverse primer, 25 nM probe, 1.1 µL diothiothreitol, 2.2 µL RT enzyme, and 4 µL template. Negative and positive controls were used in each run. All samples were run in duplicate, apart from the reproducibility analyses, which were run in triplicate with 3 different master mixes, for a total of 9 runs. Droplets were generated using a Bio Rad QX200 droplet generator and read after the PCR run using a Bio Rad QX200 droplet reader.

### RT-SIBA

RT-SIBA was performed for the wastewater samples collected in December 2021 using the Aidian SIBA protocol for SARS-CoV-2. In addition, the sensitivity of the method was determined using the SARS-CoV-2 synthetic RNA control. First, a master mix was prepared by combining 15.2 µL of the RT-SIBA A-MIX, 15.2 µL of the RT-SIBA B-MIX, and 7.6 µL of the Oligomix 1 High SYBR 1.0. Then, 0.43 µL of magnesium acetate (Sigma-Aldrich) was added to the RNA template, after which 5 µL of the RNA template was added to the master mix. A positive RNA control, a negative control for RNA extraction, and an RT-SIBA negative control were run on each plate. The SIBA reaction was run on the Bio Rad CFX96 Real-Time PCR detection system. The cycling conditions are presented in Table S6 in Multimedia Appendix 1. All the samples were run in triplicate.

### Statistical Analysis

All statistical analyses were performed on IBM SPSS software (version 28.0.1.0; 142). Statistical significance was tested using *P* values calculated with the Kruskal-Wallis H test. Correlations between SARS-CoV-2 GC numbers in wastewater and the incidence of COVID-19 cases were evaluated using the Kendall rank correlation coefficient.

### Ethical Considerations

This research did not involve human subjects. The population-level COVID-19 case data were retrieved from the National Infectious Diseases Register of Finnish Institute for Health and Welfare. The population-level case data are publicly available. The wastewater data are reported at the population level. The authors of this study are committed to following the ethical guidelines of the Finnish National Board on Research Integrity.

## Results

### RT-qPCR

The 95% LOD for the RT-qPCR was determined for each of the assays using the standard series of SARS-CoV-2 synthetic RNA control and the method described by Stokdyk et al [30]. The LOD was 18.4 GC/µL for the TaqMan N1 assay, 19.9 GC/µL for the TaqMan N2 assay, 77.6 GC/µL for the QuantiTect N1 assay, and 80.7 GC/µL for the QuantiTect N2 assay. Samples were regarded as positive if the Ct value of the sample was below 40. TaqMan RT-qPCR detected SARS-CoV-2 in 72% (n=36) of the wastewater samples, and QuantiTect RT-qPCR did so in only 20% (n=10) of the samples (Table 1). Notably, only 2 of the samples that were positive with TaqMan had SARS-CoV-2 GCs over the LOD. With QuantiTect RT-qPCR, none of the positive samples had GCs over the LOD.

The sensitivity of the N1 and N2 gene regions were compared. For both RT-qPCR methods, the N1 gene region was more sensitive. The LOD was lower for the N1 assay than for the N2 assay for both RT-qPCR kits. For TaqMan, 72% (n=36) of the samples were positive with the N1 region, whereas only 28% (n=14) were positive with the N2 region. Yet, none of the samples that were detected positive using N1 and TaqMan had GCs over the LOD, whereas with N2 TaqMan, 2 samples had GCs over the LOD. For QuantiTect, using the N1 gene region resulted in 20% (n=10) of the samples being positive, and 6% (n=3) with the N2 region. The N1 gene region was more sensitive in all incidence sample groups (Table 1). There was no statistically significant difference in the GC numbers detected by the 2 gene targets within the RT-qPCR assays (*P*=.17).

**Figure 1:**
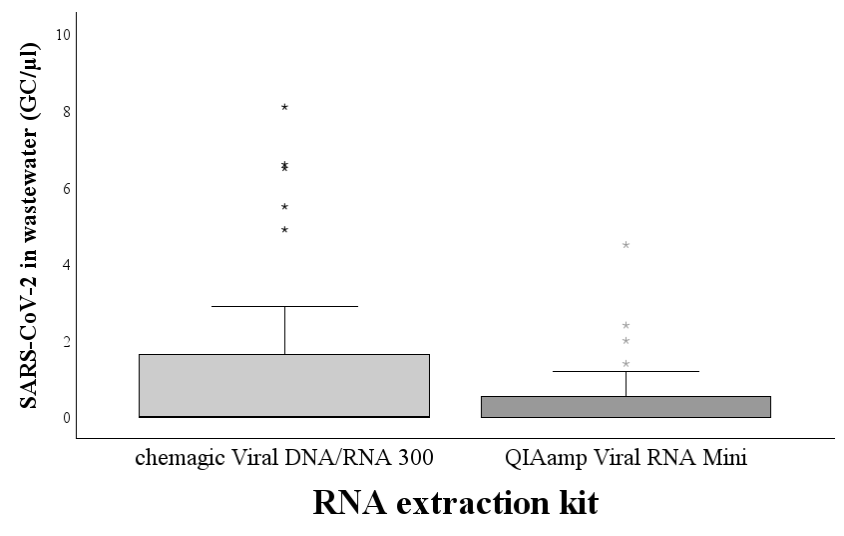
The difference in SARS-CoV-2 GC numbers per µL of sample between the PerkinElmer chemagic Viral DNA/RNA 300 and Qiagen QIAamp Viral RNA mini kit. The difference was observed using the TaqMan Fast Virus 1-step RT-qPCR (*P*=.04). GC: gene copy.

The effect of the RNA extraction method was also studied by analyzing the February and March samples with the RNA extraction kits manufactured by chemagic and Qiagen. With the TaqMan RT-qPCR, 40% (n=8) of the samples extracted with chemagic were positive and 35% (n=7) of those extracted with Qiagen were positive. The GC numbers detected from the samples extracted with chemagic were slightly higher than for those extracted with Qiagen (Figure 1). The result was statistically significant (*P*<.001). With the QuantiTect RT-qPCR, 10% (n=2) of the samples were positive using both extraction kits. There was no statistically significant difference in the GC numbers detected by the QuantiTect RT-qPCR using the 2 extraction kits (*P*=.67)

To assess the repeatability (intraassay variability) and reproducibility (interassay variability) of the most sensitive RT-qPCR method, 2 samples were analyzed using 9 replicates. The samples were collected during low incidence of COVID-19 (20 cases per 100,000 persons and 25 cases per 100,000 persons) so that variability could be assessed from samples that contained a low number of SARS-CoV-2 GCs. The results of the TaqMan RT-qPCR showed high variability (Table 2).

**Table 1:** The proportion of positive samples (%) indicated by the test methods and gene targets in wastewater samples in different incidence sample groups. The low category includes 3 samples that were collected when the incidence of COVID-19 was 0 (total=50, low incidence=23, medium incidence=14, and high incidence=13).

Test methods and gene targets	Total (%)	Incidence of COVID-19		
		Low (%)	Medium (%)	High (%)
TaqMan RT-qPCR^a^ N1	72	43	93	100
N2	28	4	36	62
QuantiTect RT-qPCR N1	20	9	21	38
N2	6	0	0	23
RT-ddPCR^b^ N1	80	30	100	100
E	86	43	100	100

^a^RT-qPCR: quantitative reverse transcriptase–polymerase chain reaction.

^b^RT-ddPCR: reverse transcriptase–droplet digital polymerase chain reaction.

**Table 2:** The variability of the TaqMan quantitative reverse transcriptase–polymerase chain reaction (RT-qPCR) and reverse transcriptase–droplet digital polymerase chain reaction (RT-ddPCR) was presented separately for the chemagic and Qiagen RNA extraction kits and N1, N2, and E assays.

Extraction kit and gene assay				Intraassay variability (%)		Interassay variability (%)
**TaqMan RT-qPCR**						
	**Chemagic**					
		N1	56		70	
		N2	65		46	
	**Qiagen**					
		N1	73		30	
		N2	75		36	
**RT-ddPCR**						
	**Chemagic**					
		N1	47		11	
		E	37		21	
	**Qiagen**					
		N1	29		7	
		E	29		13	

For the QuantiTect RT-qPCR, the template volume used was initially 2 µL/reaction. In addition, the manufacturer recommended 5 µL of template per reaction, which was also the template volume for the TaqMan RT-qPCR. The effect of the template volume used was studied using the samples collected in December 2021. The 2 µL/reaction template volume resulted in 40% (n=4) of the samples being positive and an average of 1.1 SARS-CoV-2 GC/µL. The 5 µL/reaction template volume resulted in 10% (n=1) of the samples being positive and an average of 0.2 SARS-CoV-2 copies/µL, showing that increasing the template volume negatively affected the detection of SARS-CoV-2.

**Figure 2:**
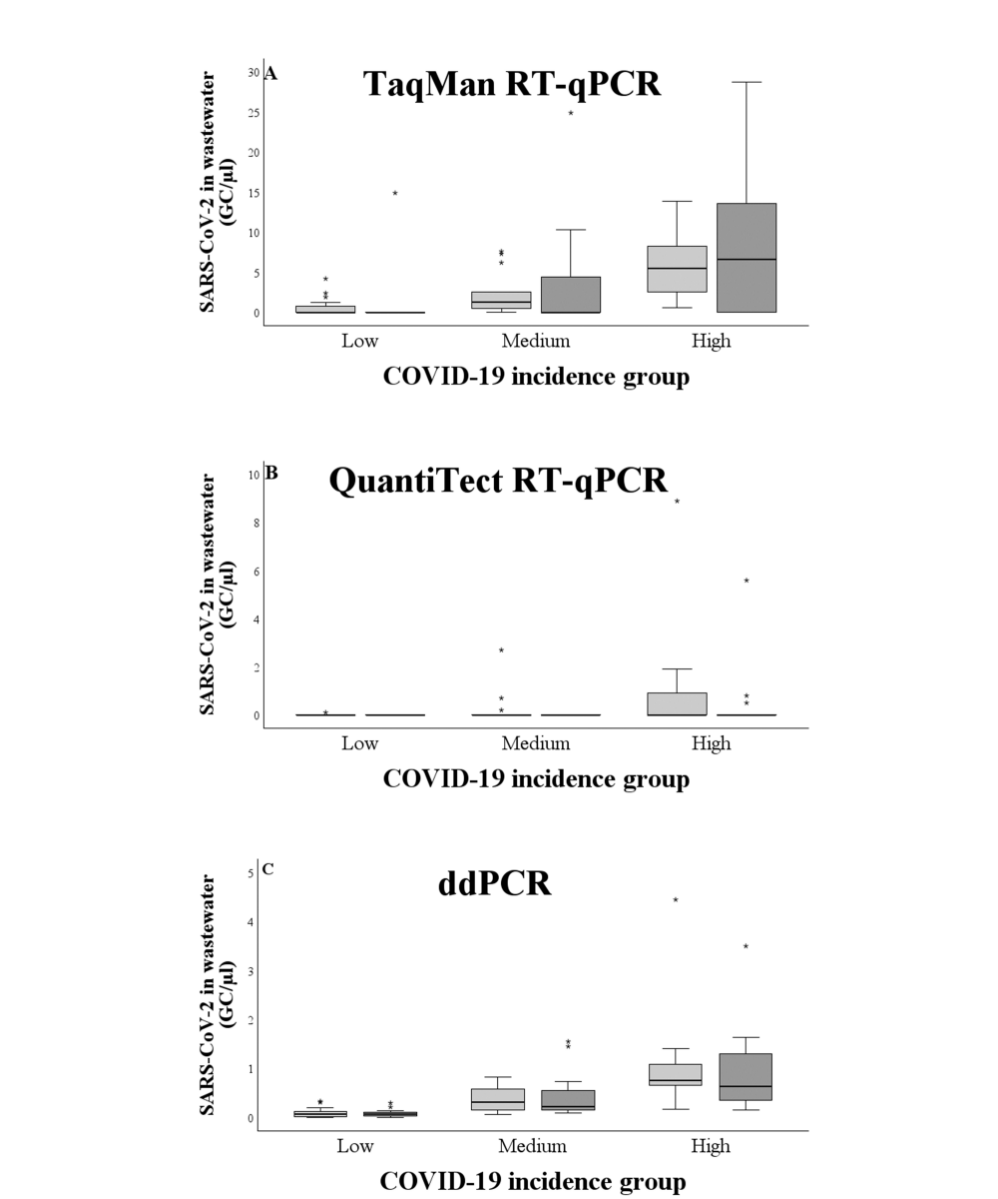
The GC numbers of SARS-CoV-2 in different COVID-19 incidence groups with both RT-qPCR kits, RT-ddPCR, and gene targets. (A) The data set of TaqMan RT-qPCR N1 (light gray) and N2 assay (dark gray), (B) the data set of QuantiTect RT-qPCR N1 (light gray) and N2 assay (dark gray), and (C) the data set of RT-ddPCR N1 (light gray) and E assay (dark gray). The differences between the incidence groups were significant for all methods (*P*<.001 for the TaqMan RT-qPCR and RT-ddPCR, *P*<.03 for the QuantiTect RT-qPCR). GC: gene copy; RT-ddPCR: reverse transcriptase–droplet digital polymerase chain reaction; RT-qPCR: quantitative reverse transcriptase–polymerase chain reaction.

A positive association was seen between the incidence of COVID-19 and the percentage of SARS-CoV-2-positive wastewater samples (Table 1). With the TaqMan RT-qPCR assay, 43% (n=10) of the low-, 93% (n=13) of the medium-, and 100% (n=13) of the high-incidence samples were positive. The same trend was seen in the QuantiTect RT-qPCR assay, with 9% (n=2) of the low-, 21% (n=3) of the medium-, and 38% (n=5) of the high-incidence samples being positive. In addition, a positive association was found between the incidence of COVID-19 and the GC numbers of SARS-CoV-2 in wastewater (Figure 2). For the TaqMan RT-qPCR assay, the mean GC/µL was 0.6 (range 0-14.9) for the low-, 3.0 (range 0-24.9) for the medium-, and 7.0 (range 0-28.7) for the high-incidence sample groups. For the QuantiTect RT-qPCR assay, the mean GC/µL was 0.0 (range 0-0.1) for the low-, 0.1 (range 0-2.7) for the medium-, and 0.4 (range 0-8.9) for the high-incidence sample groups. The differences between the GCs of different incidence groups were statistically significant for the TaqMan RT-qPCR (*P*<.001) and for the QuantiTect RT-qPCR (*P*=.03). Notably, in almost all the samples of our study, the calculated GCs were under the LOD.

**Figure 3:**
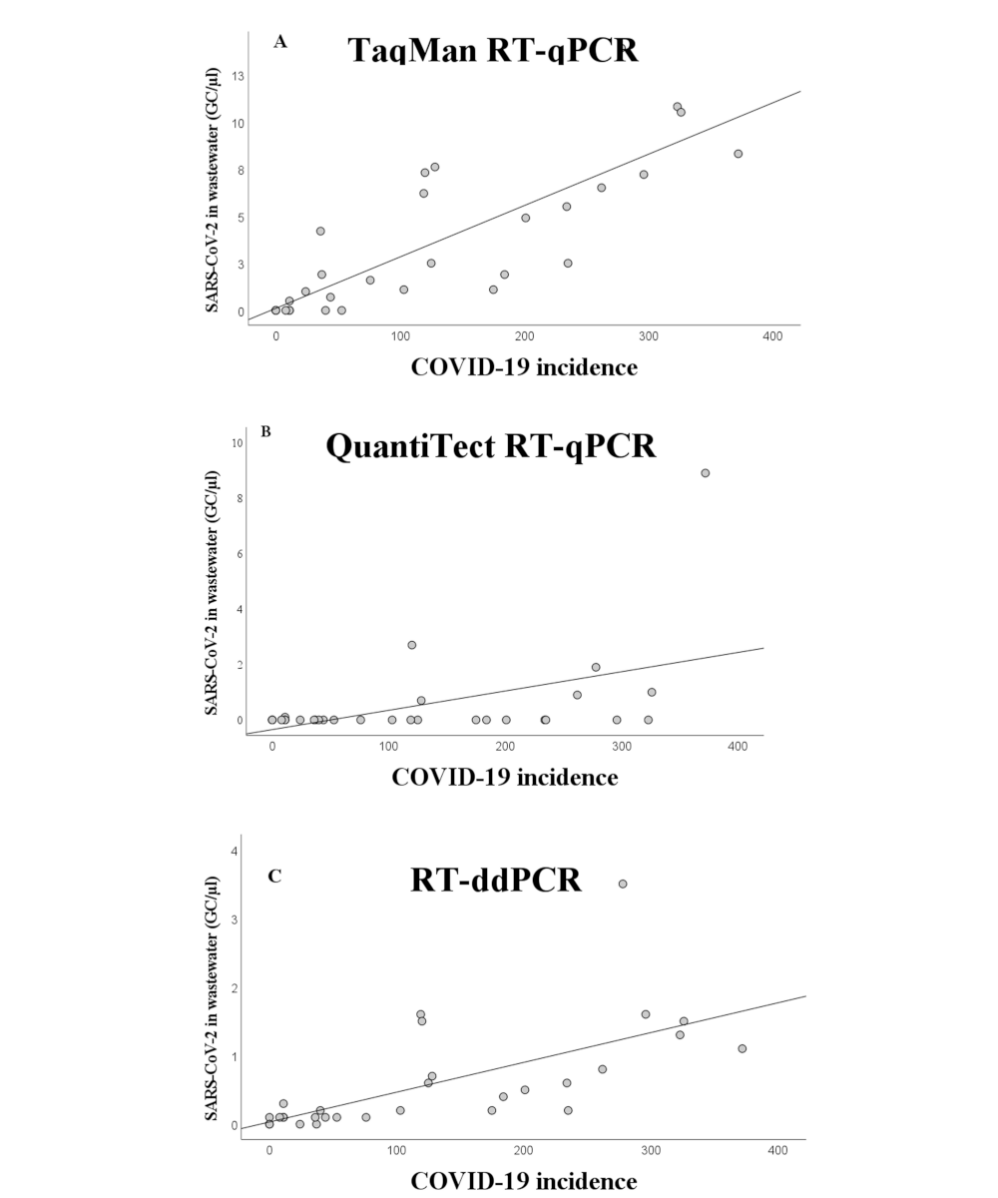
Correlation between the SARS-CoV-2 GC numbers in wastewater and COVID-19 incidence. (A) For the TaqMan RT-qPCR, the Kendall rank correlation coefficient was 0.697 (*P*<.001); (B) for the QuantiTect RT-qPCR, it was 0.351 (*P*=.02); and (C) for the RT-ddPCR, it was 0.629 (*P*<.001). The correlation was calculated using the most sensitive gene target of each method, meaning that the TaqMan and QuantiTect RT-qPCR GCs included the test results detected with the N1 assay and the RT-ddPCR GCs with the E assay. GC: gene copy; RT-ddPCR: reverse transcriptase–droplet digital polymerase chain reaction; RT-qPCR: quantitative reverse transcriptase–polymerase chain reaction.

Furthermore, the Kendall rank correlation coefficient (CC) was calculated for the SARS-CoV-2 GCs in wastewater detected by each method and the incidence of COVID-19 (Figure 3). For the TaqMan RT-qPCR, there was a positive correlation, at CC=0.697 (*P*<.001). As most of the samples were identified as negative by the QuantiTect RT-qPCR, the correlation was not as clear (CC=0.351, *P*=.02).

### RT-ddPCR

The detection limit for RT-ddPCR was determined to be 0.06 GC/µL using the standard series of SARS-CoV-2 synthetic RNA control. Out of all the wastewater samples, 86% (n=43) were positive by RT-ddPCR.

The effects of the gene target and the RNA extraction method used were studied. The E gene assay detected a slightly higher number of positive samples (43/50, 86%) than the N1 gene assay (40/50, 80%; Table 1). There was no statistically significant difference between the SARS-CoV-2 GCs of the 2 tested gene targets. Furthermore, there was no statistically significant difference in GCs detected between the chemagic and Qiagen extraction kits.

To assess the repeatability (intraassay variability) and reproducibility (interassay variability) of the detection, 2 wastewater samples were analyzed using 9 replicates. The samples were collected during low incidence of COVID-19 (20 cases per 100,000 persons and 25 cases per 100,000 persons) so that variability could be assessed from samples that contained a low amount of SARS-CoV-2. The results of RT-ddPCR showed high variability (Table 2).

A positive association was found between the incidence of COVID-19 and the percentage of SARS-CoV-2-positive wastewater samples (Table 1). From the low-incidence group of samples, 43% (n=10) were positive, whereas all samples from the medium- and high-incidence groups were positive (Table 1). Furthermore, a positive association was observed between the incidence of COVID-19 and the SARS-CoV-2 GCs in wastewater (Figure 2). The mean GC number was 0.08 (0-0.3) for the low-, 0.4 (0.1-1.6) for the medium-, and 1.0 (0.2-4.5) for the high-incidence groups. The results were statistically significant (*P*<.001). Furthermore, the SARS-CoV-2 GC numbers in wastewater correlated with the incidence of COVID-19 (CC=0.629, *P*<.001; Figure 3).

### Correlation Between the Methods

**Figure 4:**
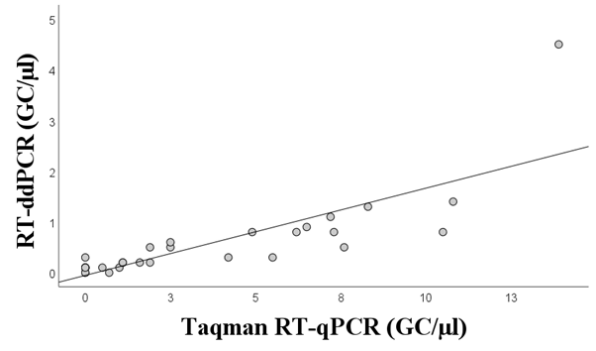
Correlation between the SARS-CoV-2 GC numbers in wastewater detected by TaqMan RT-qPCR and RT-ddPCR. The Kendall rank correlation coefficient was 0.783 (*P*<.001). The test results analyzed using the N1 assay were included. GC: gene copy; RT-ddPCR: reverse transcriptase–droplet digital polymerase chain reaction; RT-qPCR: quantitative reverse transcriptase–polymerase chain reaction.

The correlation between the methods was investigated using wastewater results. A positive correlation was observed between the TaqMan RT-qPCR and RT-ddPCR assays (CC=0.783, *P*<.001; Figure 4). However, the quantification of SARS-CoV-2 GC numbers in the wastewater samples was much higher with the TaqMan RT-qPCR than with the RT-ddPCR method. No correlation was found between the QuantiTect RT-qPCR assay and other methods due to the low positivity rate of the QuantiTect RT-qPCR assay.

### RT-SIBA

The detection limit for RT-SIBA was determined to be 10 GC/µL using the standard series of SARS-CoV-2 synthetic RNA control. On average, 10 GC/µL of the template was detected in 22 minutes, 100 GC/µL in 19 minutes, 1000 GC/µL in 15 minutes, and 10,000 GC/µL in 12 minutes. The wastewater samples collected in December 2021 were analyzed by RT-SIBA assay. All these samples were positive for SARS-CoV-2.

## Discussion

### Principal Findings

This study was conducted to compare different methodologies in the analysis of SARS-CoV-2 RNA in wastewater. Our results showed that RT-ddPCR was more sensitive to detect SARS-CoV-2 in wastewater compared to RT-qPCR. RT-ddPCR had the highest positivity rate (26/30), and its limit of detection was the lowest (0.06 GC/µL). Thus, the use of RT-ddPCR in the wastewater surveillance of SARS-CoV-2 should be considered, especially if the amount of SARS-CoV-2 circulating in the population is low. Still, we achieved the best correlation between COVID-19 incidence and SARS-CoV-2 GC number in wastewater using RT-qPCR (CC=0.697, *P*<.001). Furthermore, changes in the analysis pipeline were shown to affect the results. There was a significant difference in sensitivity between the TaqMan RT-qPCR and QuantiTect RT-qPCR. In addition, for RT-qPCR, both the choice of the gene target and the RNA extraction method affected the results. As the compatibility of the RNA extraction method, the RT-qPCR kit, and the target gene assay affected the results, all the analysis steps must be optimized for wastewater surveillance in the specific conditions of a study.

### Comparison of RT-qPCR and RT-ddPCR

Based on our study, the most sensitive method for detecting SARS-CoV-2 in wastewater was RT-ddPCR, since its limit of detection was lower than that of RT-qPCR and it had a higher positivity rate. Furthermore, it had a lower variance of results. This is in line with most of the previous studies [13-16,32]. However, another study reported that RT-qPCR was more sensitive in detecting the virus from postgrit solids and primary clarified sludge because of its lower LOD and detected copy numbers [12]. Some studies have found the methods to be similar in sensitivity [17,20].

All tested methods showed a positive association between the incidence of COVID-19 and the amount of SARS-CoV-2 detected in wastewater. The strongest correlation was found using the TaqMan RT-qPCR with the N1 gene as the detection target. The use of the N2 gene resulted in a weaker correlation, possibly due to its lower sensitivity. Although RT-ddPCR was the most sensitive method, its correlation with the incidence of COVID-19 was slightly weaker than that of the TaqMan RT-qPCR N1 gene assay. Still, it should be considered that almost all the samples were under the LOD of RT-qPCR, and thus, the quantification of the copy numbers by RT-qPCR might not be reliable and thus the correlation might not be reliable. There was no major difference in the correlation coefficients of RT-ddPCR between the 2 gene targets. The correlation between the QuantiTect RT-qPCR assays and the incidence of COVID-19 was weak due to low sensitivity. However, these results show that wastewater surveillance can be used to estimate the circulation of SARS-CoV-2 in a population with optimized PCR-based assays.

Most of the previous methodology papers comparing RT-ddPCR and RT-qPCR only used 1 RT-qPCR kit. In this study, we wanted to study the differences between kits using the same oligonucleotides. The TaqMan RT-qPCR had a significantly lower LOD (18.4 GC/µL for the N1 assay and 19.9 GC/µL for the N2 assay) than the QuantiTect RT-qPCR (77.6 GC/µL for the N1 assay and 80.7 GC/µL). This shows that the 2 RT-qPCR kits have significant differences in their sensitivity. This was also reflected in the analysis of the samples, as the TaqMan RT-qPCR kit showed a higher positivity rate (72% N1 gene and 20% N2 gene) than the QuantiTect RT-qPCR (20% N1 gene and 6% N2 gene). These results indicate that there were significant differences in the performance of RT-qPCR kits even though the methods use the same oligonucleotides. This could partly explain the differences between previous studies, as the studies used different RT-qPCR kits. The difference between kits and assays should always be considered in addition to the differences between RT-qPCR and RT-ddPCR when the results of different studies are compared. Furthermore, it is vital that the methods are described in detail according to the approved guidelines for a reliable comparison of the studies [33].

### Comparison of the Target Gene Assays

In addition to different PCR-based methods, different target gene assays were also compared in the study. For RT-ddPCR, the LOD was the same for both target gene assays, and the positivity rate was very similar (86% for the E assay and 80% for the N1 assay). Previously, N1 was reported to be the most sensitive gene target for RT-ddPCR [32], but it is also prone to outliers and correlates poorly with other gene targets [34]. For both RT-qPCR kits, a significant difference between the 2 target gene assays was noted. The LOD was lower for the N1 assay than the N2 assay for both TaqMan RT-qPCR and QuantiTect RT-qPCR. Furthermore, the positivity rate was also higher for the N1 assay using both RT-qPCR kits. For TaqMan, the positivity rate was 72% for the N1 assay and 20% for the N2 assay. For QuantiTect, the positivity rate was 20% for the N1 assay and 6% for the N2 assay. The RT-qPCR results are in agreement with previous studies [20,32]. In contrast, Flood et al [15] discovered N2 to perform better than N1 and E target genes. They also noted that the E gene resulted in nearly all samples being nondetects with RT-qPCR. We obtained similar results at the optimization phase of the RT-qPCR methods, and therefore, the E gene was not used in this study with RT-qPCR (data not shown). There was no statistically significant difference between the target gene assays in the quantification of the samples. It was previously found that N2 was more prone to inhibition than N1 in RT-qPCR, while a difference in inhibition was not seen between the gene targets using RT-ddPCR. RT-ddPCR has been noted to be less sensitive to inhibition than RT-qPCR, most likely due to nanodroplet quantification [20]. This could explain why studies have not found significant differences in the target gene assays with ddPCR, whereas significant differences have been noted with qPCR.

### Comparison of the RNA Extraction Methods

The 2 RNA extraction methods were compared in the study to see the effect that different assays and analysis pipelines can have on the detection of viruses in wastewater. With TaqMan RT-qPCR, using the chemagic RNA extraction kit produced higher GCs and positivity rates compared to the QIAamp RNA extraction kit. The chemagic RNA extraction concentrated samples 3-fold and the QIAamp kit 2.3-fold. Previous studies have also noted variations in the results between RNA extraction kits [18,19]. O’Brien et al [18] achieved the best result using an RNA extraction kit that had PCR inhibitor removal. Our RNA extraction methodologies did not have inhibitor removal, which could have resulted in the RT-qPCR being negatively affected by inhibition. Zheng et al [19] noted significant 10-fold differences in the sensitivities of 2 different RNA extraction kits. These results show that the whole sample processing protocol should be optimized for the sample matrix. None of the previous methodology studies used completely the same analysis pipeline, including RNA extraction kit, RT-qPCR and RT-ddPCR kits, and gene assay. This should always be considered when different studies are compared. To ensure the quality and comparability of a study, the methods as well as their quality control should be depicted in detail.

### RT-SIBA

An isothermal amplification method, RT-SIBA, was tested for the detection of SARS-CoV-2 in wastewater. The sensitivity of RT-SIBA 10 GC/µL (23 GC/reaction) was similar to that of an RT-SIBA assay used for clinical diagnostics (25 GC/reaction) [25]. The RT-LAMP method was also applied for the wastewater analysis of SARS-CoV-2. The assay was less sensitive than RT-SIBA (LOD of 76 N1 GC/reaction) [26]. RT-SIBA was used for testing 10 wastewater samples in this study, and all the tested samples were positive. The method was able to provide results quickly. RT-SIBA detected 10 GC/µL of SARS-CoV-2 in 22 minutes. Previously, RT-SIBA was reported to detect 100 GCs of influenza types A and B virus in 15 minutes [22] and 10 GCs of respiratory syncytial virus in 20 minutes [23]. These results indicate that RT-SIBA is a potential alternative for the detection of SARS-CoV-2 in wastewater. It is a viable option if results are needed quickly and presence-absence information is sufficient. In addition, the method is also easier to perform and cheaper than RT-qPCR or RT-ddPCR. It does not require complicated instruments, as the reaction is isothermal [22]. Still, more research into the use of RT-SIBA in wastewater surveillance is required, as the sample size for RT-SIBA was small in this study.

### Limitations

The aim of the study was to evaluate the performance of the methods under challenging conditions and close to the LOD of the methods. Therefore, the samples were collected when the GC number of SARS-CoV-2 in wastewater was low. Even though we were able to detect samples to be positive with RT-qPCR, the amount of the virus was under the detection limit in almost all samples, and we were not able to quantify the GCs reliably with RT-qPCR. All the samples that were detected positive were over the LOD of RT-ddPCR. This is most likely explained by the fact that the LOD was significantly lower for RT-ddPCR than RT-qPCR, showing RT-ddPCR to be more sensitive than RT-qPCR. Thus, if the amount of SARS-CoV-2 in wastewater is low, the use of RT-ddPCR should be considered.

Our study indicated that there was a variation in virus quantification between RT-ddPCR and RT-qPCR methods. According to the manufacturer, the RNA standard for SARS-CoV-2 contained 10,000 GC/µL, but according to RT-ddPCR it contained only 253 GC/µL. D’Aoust et al [12] noted that the GCs of the samples were lower according to ddPCR than to qPCR. Another study found the GCs detected by the 2 methods to be similar [20]. The GC numbers of the RNA standard might not be accurate, as RNA degrades rapidly. The standard used in the quantification of the RT-qPCR may affect its results depending on the accuracy of the standard’s reported GCs. Variations in quantification between different methods should be considered when estimating the amount of SARS-CoV-2 in wastewater. However, a linear positive correlation was observed between the results obtained by the TaqMan RT-qPCR and RT-ddPCR. The problem with differences in quantification can be minimized by following the trends and trendlines of SARS-CoV-2 in wastewater.

Both RT-ddPCR and RT-qPCR had a high variation in the quantification of SARS-CoV-2 GCs in wastewater. In the repeatability and reproducibility test, the lowest intraassay variability was 29% for RT-ddPCR and 56% for the TaqMan RT-qPCR, and the lowest interassay variability was 7% for RT-ddPCR and 30% for the TaqMan RT-qPCR. The variance differed between gene targets and RNA extraction kits, but no clear pattern was found. It is known that variation increases close to the detection limit. This may partly explain the results since the variability was tested using samples that had a very low number of SARS-CoV-2 GCs that were mostly under the limit of detection. The high variability is likely partly due to wastewater being the sample matrix and to the low GCs of the samples. The TaqMan RT-qPCR showed a low SD (approximately 15%) in a high copy number sample (RNA standard of 10,000 GC/µL). Furthermore, 1 study on SARS-CoV-2 detection in wastewater suggested variability to be most likely affected by the inhibitors present in wastewater [35]. The variability and poor reproducibility of SARS-CoV-2 wastewater detection was also noted as a frequent problem in another recent review [36]. Particularly during the low incidence of virus in the community, the estimation of virus levels in wastewater may be inaccurate.

### Conclusions

The wastewater monitoring of SARS-CoV-2 has been mostly implemented using different RT-qPCR and RT-ddPCR assays. However, wastewater is a complex sample matrix for monitoring SARS-CoV-2, compared to clinical samples. Previous comparisons of the performance of RT-qPCR and RT-ddPCR in wastewater matrix have been conflicting. Our study found that the compatibility of all analysis steps, including the RNA extraction method, RT-PCR kit, and gene assay used, as well as quantitative control, influence the performance of the tests; therefore, all analysis steps must be optimized for wastewater samples. Currently, SARS-CoV-2 is monitored mostly with RT-qPCR assays. Yet, our study, as well as most previous studies, showed RT-ddPCR to be a more sensitive assay. Thus, RT-ddPCR is a valid option for the monitoring of SARS-CoV-2 in wastewater if a low amount of SARS-CoV-2 is circulating in the population. Our study showed that isothermal amplification RT-SIBA can also be used for the detection of SARS-CoV-2 in wastewater if a qualitative result of SARS-CoV-2 is sufficient. RT-SIBA enables the detection of SARS-CoV-2 faster and with fewer resources than RT-qPCR and RT-ddPCR methods.

SARS-CoV-2 GC numbers in wastewater reflect the incidence of COVID-19 in the population. Experience with SARS-CoV-2 virus monitoring has encouraged the development of monitoring methods for other human-infecting viruses in wastewater. Wastewater surveillance has the potential to improve the health care system and public health preparedness for microbial epidemics and pandemics.

## Appendix

Multimedia Appendix 1 Additional material.

## Abbreviations

CC: correlation coefficient
GC: gene copy
LOD: limit of detection
PCR: polymerase chain reaction
RT-ddPCR: reverse transcriptase–droplet digital polymerase chain reaction
RT-LAMP: reverse transcriptase–loop-mediated isothermal amplification
RT-qPCR: quantitative reverse transcriptase–polymerase chain reaction
RT-SIBA: reverse transcription strand invasion–based amplification
WWTP: wastewater treatment plant

## Data Availability

The data sets generated or analyzed during this study are available from the corresponding author on reasonable request.
